# Supplementary material for: Analysis of PPARγ Signaling Activity in Psoriasis
Source: Int J Mol Sci. 2021 Aug 10;22(16):8603. doi: 10.3390/ijms22168603 (PMC8395241; doi:10.3390/ijms22168603)
Supplement: Supplementary file 1 [file ijms-22-08603-s001.zip › Supplemental materials_Analysis of PPARg signaling activity in psoriasis/Pathway models/Models images and html files/Model of PPARG related pathways in psoriasis (short version)/11858.html]

LXR


# Functional Class LXR

|  |  |
| --- | --- |
| URN | urn:agi-protfc:lxr |
| Total Entities | 4 |
| Connectivity | 2597 |
| Name | LXR |

---

|  |  |
| --- | --- |
| ChildConcepts | NR1H2 |
|  | NR1H4 |
|  | NR1H3 |
|  | m\_Nr1h5 |

---

|  |  |
| --- | --- |
| Pathway | Amyloid beta and APP Intracellular Transport in Alzheimer Disease |
|  | Neighbors of LXR |
|  | Model of PPARG related pathways in psoriasis (short version) |
|  | before laser treatment |
|  | PPARG signaling after laser treatment |

---

|  |  |
| --- | --- |
| MedScan ID | 12000141 |

---

|  |  |
| --- | --- |
| Alias | LXRs |
|  | liver X receptors |
|  | liver X receptor |
|  | LXR |

---
